# Supplementary material for: MFN1 and MFN2 Are Dispensable for Sperm Development and Functions in Mice
Source: Int J Mol Sci. 2021 Dec 16;22(24):13507. doi: 10.3390/ijms222413507 (PMC8707932; doi:10.3390/ijms222413507)
Supplement: Supplementary file 1 [file ijms-22-13507-s001.zip › ijms-1500185-SI.pdf]

Table S1. Transmission efficiency of mutant alleles by *Prm1-Cre* from Jackson Lab.

Floxed alleles	NO. of males	NO. of litters	NO. of pups	*f*/+ pups	Del/+ pups	Transmission Del/+ allele
Mfn1$^{f/f}$; *Prm1-Cre*	2	4	35	8	27	77%
Mfn2$^{f/f}$; *Prm1-Cre*	3	6	50	24	26	52%

Table S2. Primers used in genotyping.

Gene name	Forward primers (5' to 3')	Reverse primers (5' to 3')	Size of Amplicon
Prm1-Cre (Jackson Laboratory)	GCGGTCTGGCAGTA AAAACTATC	GTGAAACAGCATTGCT GTCACTT	100bp
Prm1-Cre (RIKEN BRC)	GTTTCACTGGTTATG CGGCGG	TTCCAGGGCGCGAGTT GATAG	450bp
Mfn1-floxed	TTGGTAATCTTTAGC GGTGCTC	AGCAGTTGGTTGTGTG ACCA	Homozygous:450bp Wildtype:350bp
Mfn1-delete	TTGGTAATCTTTAGC GGTGCTC	TTAAAGACACGGCTAA TGGCAG	Cre excised:325bp
Mfn2-floxed	GAAGTAGGCAGTCT CCATCG	AACATCGCTCAGCCTG AACC	Homozygous:180bp Wildtype:145bp
Mfn2-delete	GAAGTAGGCAGTCT CCATCG	ACATCACCCTGAAGCC TGGTTT	Cre excised:350bp

Table S3. Primers used in qPCR.

Gene name	Forward primers (5' to 3')	Reverse primers (5' to 3')
Mfn1	AGCGGGATTGGTCACACAAC	CCTTCGGTCATAAGGTAGGCTT
Mfn2	GTGGGCTGGAGACTCATCG	CTCACTGGCGTATTCCACAA
Gapdh	AGGTCGGTGTGAACGGATTTG	TGTAGACCATGTAGTTGAGGTCA

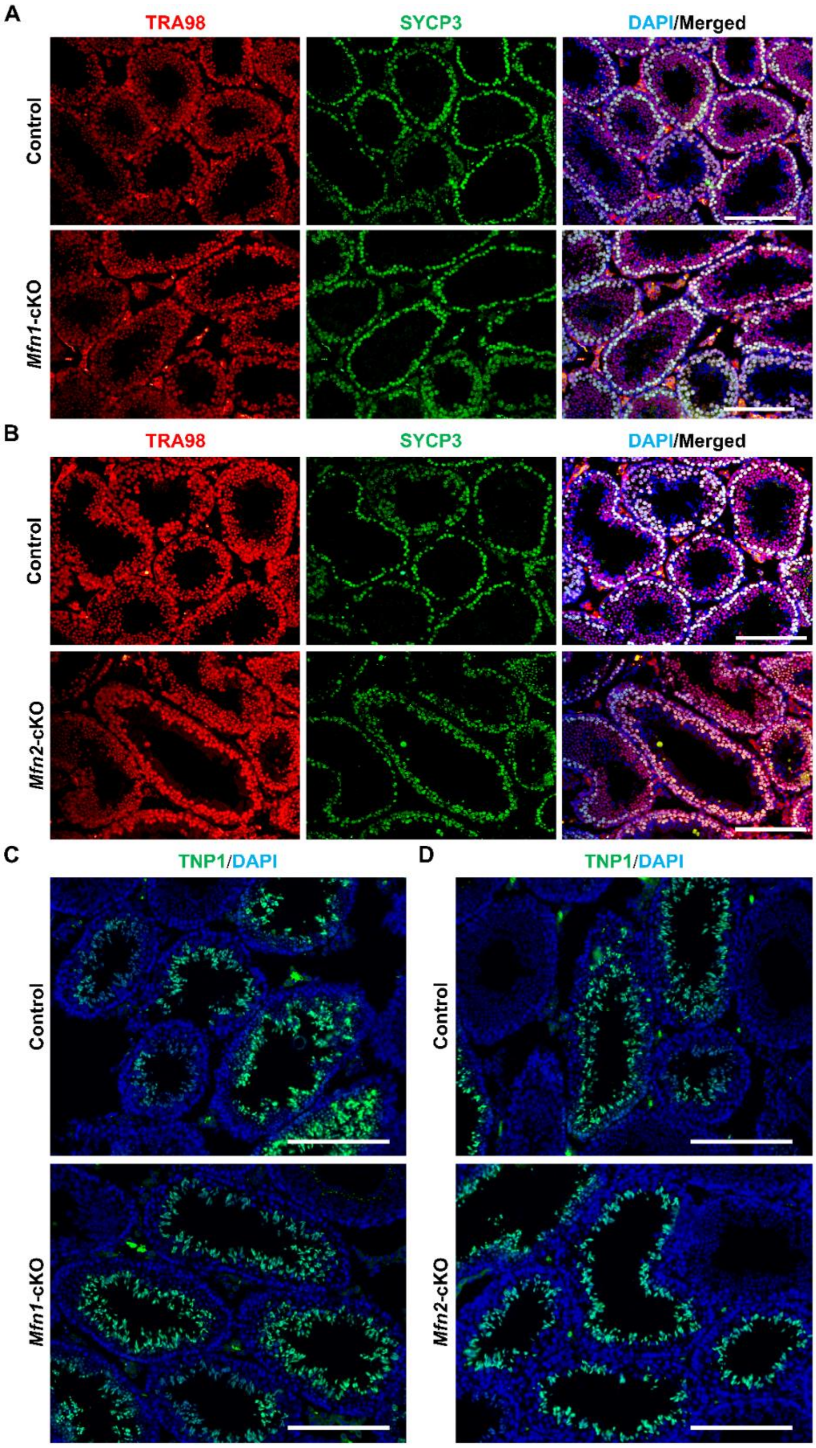

Figure S1. Either *Mfn1* or *Mfn2* deletion does not affect sperm development. (A-B) The development of pan-germ cells (indicated by TRA98 staining) and spermatocytes (labeled by SYCP3 staining) was examined by IHF on *Mfn1*-cKO **(A)** or *Mfn2*-cKO **(B)** and control testis sections from 6-week mice. Scale bars: 200 μm. **(C-D)** The elongated spermatids were examined by IHF with a TNP1 antibody, counterstained with DAPI on *Mfn1*-cKO **(C)** or *Mfn2*-cKO **(D)** and control testis sections from 6-week mice. Scale bars: 200 μm.

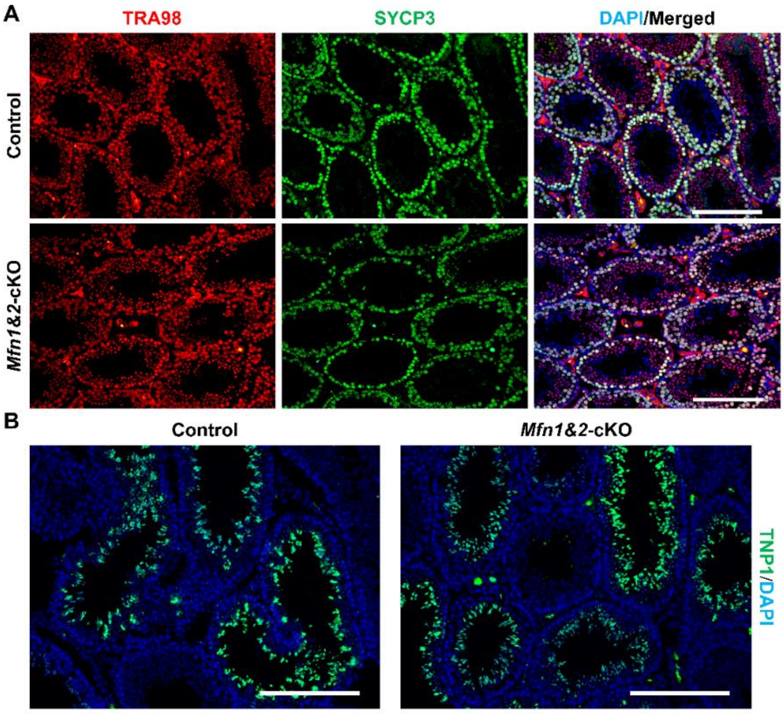

Figure S2. Combined deletion of *Mfn1* and *Mnf2* in sperm does not affect sperm development. (A) The development of pan-germ cells (indicated by TRA98 staining) and spermatocytes (labeled by SYCP3 staining) was examined by IHF on *Mfn1&2*-cKO and control testis sections from 6-week mice. **(B)** The elongated spermatids were examined by IHF with a TNP1 antibody, counterstained with DAPI on *Mfn1&2*-cKO and control testis sections from 6-week mice. **(A-B)** Scale bars: 200 μm.

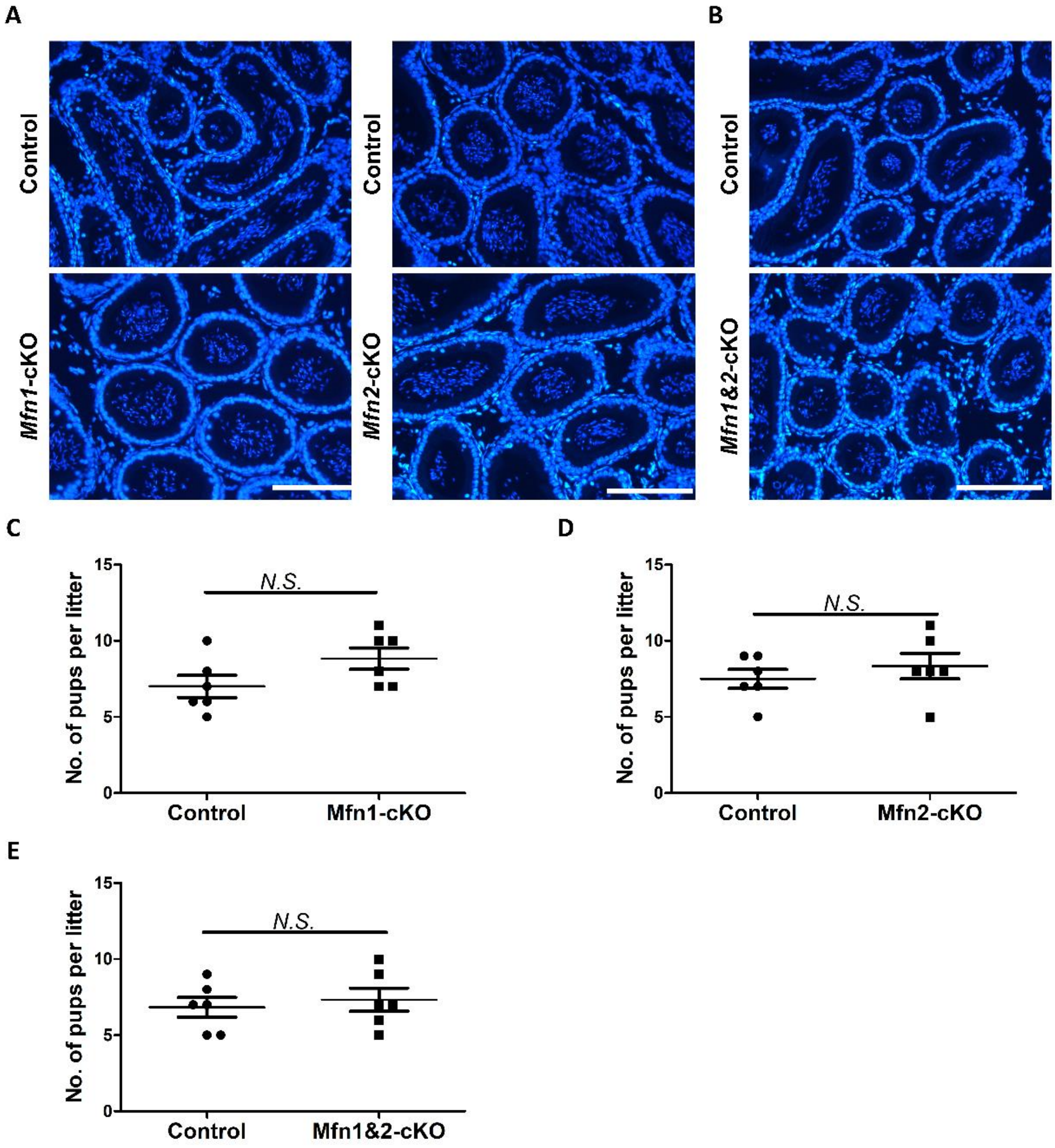

Figure S3. Partial deletion of *Mfn1* and/or *Mfn2* in sperm by *Prm1-Cre* from Jackson lab does not affect sperm development and male fertility. (A) DAPI staining in epididymides from control and *Mfns*-cKO mice at 14-week. (B) DAPI stained sections of epididymides from control and *Mfn1&2*-cKO mice at 8-week, (A–B) Scale bars, 200 μm. (C) Averaged pup numbers per litter were calculated based on 6 litters from 3 control and 3 *Mfn1*-cKO male mice. Each male was separately bred with 2 wildtype females for at least 2 months. (D) Averaged pup numbers per litter were calculated based on 6 litters from 3 control and 3 *Mfn2*-cKO male mice. Each male was crossed with 2 wildtype females for over 2 months. (E) Averaged pup numbers per litter were calculated based on 6 litters from 3 control and 3 Mfn1&2-cKO male mice. Each male was bred with 2 wildtype females for at least 2 months. (C–E) Data are presented as mean ± SEM. N.S.: no significance.
